# CRISPR screening identifies CDK12 as a conservative vulnerability of prostate cancer

**DOI:** 10.1038/s41419-021-04027-6

**Published:** 2021-07-27

**Authors:** Hanqi Lei, Zifeng Wang, Donggen Jiang, Fang Liu, Meiling Liu, Xinxing Lei, Yafei Yang, Bin He, Min Yan, Hai Huang, Quentin Liu, Jun Pang

**Affiliations:** 1 Department of Urology, Kidney and Urology Center, Pelvic Floor Disorders Center, The Seventh Affiliated Hospital, Sun Yat-sen University, Shenzhen, China; 2 Department of Urology, Sun Yat-sen Memorial Hospital of Sun Yat-sen University, Guangzhou, China; 3 Sun Yat-sen University Cancer Center, State Key Laboratory of Oncology in South China, Collaborative Innovation Center for Cancer Medicine, Guangzhou, China

**Keywords:** Targeted therapies, Prostate cancer

## Abstract

Androgen receptor (AR) signaling inhibitors provide limited survival benefits to patients with prostate cancer (PCa), and worse, few feasible genomic lesions restrict targeted treatment to PCa. Thus, a better understanding of the critical dependencies of PCa may enable more feasible therapeutic approaches to the dilemma. We performed a kinome-scale CRISPR/Cas9 screen and identified cyclin-dependent kinase 12 (CDK12) as being conservatively required for PCa cell survival. Suppression of CDK12 by the covalent inhibitor THZ531 led to an obvious anti-PCa effect. Mechanistically, THZ531 downregulated AR signaling and preferentially repressed a distinct class of CDK12 inhibition-sensitive transcripts (CDK12-ISTs), including prostate lineage-specific genes, and contributed to cellular survival processes. Integration of the super-enhancer (SE) landscape and CDK12-ISTs indicated a group of potential PCa oncogenes, further conferring the sensitivity of PCa cells to CDK12 inhibition. Importantly, THZ531 strikingly synergized with multiple AR antagonists. The synergistic effect may be driven by attenuated H3K27ac signaling on AR targets and an intensive SE-associated apoptosis pathway. In conclusion, we highlight the validity of CDK12 as a druggable target in PCa. The synergy of THZ531 and AR antagonists suggests a potential combination therapy for PCa.

## Introduction

The primary therapy for prostate cancer (PCa) is targeting androgen receptor (AR) signaling, while the disease inevitably progresses to castration-resistant prostate cancer (CRPC). Next-generation AR signaling inhibitors have significantly improved the survival of patients with CRPC, but further resistance remains an issue [1–3]. For targeted therapy, large-scale genomic analyses have discovered genetic drivers (ETS fusions, CDKN2A loss, PTEN, RB1, and SPOP mutations, etc.) and delineated distinct molecular subtypes of PCa [4–6], while few genetic abnormalities are being actively translated into promisingly druggable targets. Therefore, a better understanding of the critical dependencies of PCa may enable more feasible therapeutic approaches to the dilemma.

Compared with shRNA- or siRNA-based genetic dependency screens, CRISPR/Cas9 technology minimizes off-target effects, maintains higher efficiency and identifies more fitness genes [7, 8]. Current genome-scale CRISPR/Cas9 screens have been successfully used to identify genes required for cancer cell survival as candidate targets [9, 10], while their further synergy with existing medicine has been less studied. Relatively, studies used CRISPR/Cas9 screens and focused on specific genes of “synthetic lethality” for clinical drugs, but neglected cancer dependencies under medicated stress [11, 12]. This screening strategy provides potential targets for synergy, while attenuates the applicable value for monotherapy to a certain extent. To our knowledge, we first identified cyclin-dependent kinase 12 (CDK12) as conservatively required for PCa cells under both normal and AR antagonism stress conditions, suggesting that CDK12 inhibition may confer synergistic anti-PCa and AR antagonism properties.

CDK12 primarily regulates transcription elongation by phosphorylating serine-2 (S2) of the C-terminal domain (CTD) of RNA polymerase II (RNAPII) [13] and plays an essential role in DNA damage repair (DDR), especially homologous recombination (HR) [14, 15]. CDK12 has been identified to function as an oncogenic driver in several malignancies [16–18]. Notably, a CDK12 somatic loss mutation induces a novel, genetically unstable subtype of advanced CRPC [19–21]. Therefore, the role of CDK12 in PCa requires further elaboration.

Here, we report that CDK12 is a conservative target of PCa. The underlying mechanisms that mediate the conservative vulnerability of CDK12 may be driven by its preferential repression of basic survival pathways and super-enhancer (SE) associated oncogenes. Furthermore, we validated the striking synergy between CDK12 inhibition and AR antagonism. H3K27ac alteration on AR signaling may account for the synergistic effect.

## Materials and methods

### Pooled CRISPR screen

For the design of the kinome CRISPR library, 5157 gRNAs targeting 507 human kinases were selected. Then, oligo sgRNA sequences with flanking adaptors were synthesized by Synbio Technologies (Monmouth Junction, NJ, USA). The oligo pool was amplified via PCR using primers with the lenti‐CRISPR V2 vector, and then the product was subsequently inserted into the lenti‐CRISPR V2 vector using the Gibson Assembly.

The kinome CRISPR library was introduced into C4–2 cells by lentiviral transduction. After 7 days of puromycin selection, all remaining cells were divided into input, normal (DMSO), and AR antagonism (enzalutamide 10–25 μM) groups. PCa cells were then collected for DNA extraction on the 21st and 28th days. Changes in library representation were determined by quantification of the barcode identifiers present in each gRNA vector through next-generation sequencing by Novogene using the Illumina NovaSeq 6000 platform. Raw read count data were acquired and processed with the model-based analysis of genome-wide CRISPR/Cas9 knockout (MAGeCK) software to prioritize sgRNAs and genes, and the results are presented as robust rank aggregation (RRA) scores.

### Human cell lines

The cell lines used in this study were maintained in a 37 °C and 5% CO_2_ incubator. LNCaP, C4–2, 22RV1, and DU145 cells were cultured in RPMI-1640 medium (Gibco), while PC3 cells were cultured in DMEM (Gibco). All media were supplemented with 10% fetal bovine serum (FBS) (Gibco), glutamine and penicillin/streptomycin. Mycoplasma contamination was excluded via a PCR-based method.

### Compounds and antibodies

THZ531 (HY-103618), enzalutamide (HY-70002), apalutamide (HY-16060), and bicalutamide (HY-14249) were purchased from MCE. Antibodies against CDK12 (ab37914) were purchased from Abcam. Additional antibodies against CDK12 (PAB39156) were purchased from Bioswamp. Antibodies against phospho-CTD-RNAPII-S2 (Cat# 041571-l, lot# 3023013), CTD-RNAPII (Cat# 05–623–25UG, lot# Q2925497), AR (Cat# 5153T), and H3K27ac (Cat# 8173T, lot# 6) were purchased from Cell Signaling Technology. Antibodies against GRIN3A (Cat# bs12100R) were purchased from Bioss.

### Bioinformatics analysis tools

We downloaded datasets for PCa cell lines (LNCaP, DU145, and 22RV1) from the Project Score database (https://score.depmap.sanger.ac.uk/) and analyzed them in terms of the whole genome. Raw read count data were acquired and processed with MAGeCK to prioritize sgRNAs and genes, and the results are presented as beta scores, whose values are <0 and represent fitness effects. We obtained datasets regarding SE-associated genes identified in LNCaP cells treated with R1881 that had been processed by SEdb (http://www.licpathway.net/sedb/, GSE73994). The associations of disease-free survival (DFS), recurrence and PCa status with genes were processed with the following websites:

CANCERTOOL: http://web.bioinformatics.cicbiogune.es/CANCERTOOL/index.html,

THPA: https://www.proteinatlas.org,

GEPIA: http://gepia.cancer-pku.cn/.

### Tissue microarray (TMA) analysis

A commercially available human PCa TMA (HProA150CS01, Outdo Biotech, Shanghai, China) including samples from 100 patients (150 PCa and 50 normal prostate tissue specimens) who underwent radical prostatectomy was used to analyze CDK12 protein expression. Two pathologists without knowledge of patient characteristics independently assessed the immunohistochemistry (IHC) score. The immunostaining score was calculated as the percentage score × the intensity score.

### Individual plasmid construction and virus production

The lenti‐CRISPR V2 vector was used to generate the sgCDK12 and sgGRIN3A constructs. The sgNT control construct served as the empty control vector.

The sequences of the sgRNAs are listed below:

lentiCRISPRv2-sgCDK12#1-F: CACCGGCAAGAAGGACGGGAGTGG

lentiCRISPRv2-sgCDK12#1-R: AAACCCACTCCCGTCCTTCTTGCC

lentiCRISPRv2-sgCDK12#2-F: CACCGGAGACTGATGACTATGGGA

lentiCRISPRv2-sgCDK12#2-R: AAACTCCCATAGTCATCAGTCTCC

lentiCRISPRv2-sgGRIN3A#1-F: CACCGAGAAGTTTTGCTGTCACGG

lentiCRISPRv2-sgGRIN3A#1-R: AAACCCGTGACAGCAAAACTTCTC

lentiCRISPRv2-sgGRIN3A#2-F: CACCGCGACATGGAAAGTATCCGG

lentiCRISPRv2-sgGRIN3A#2-R: AAACCCGGATACTTTCCATGTCGC

### Protein lysate preparation and western blotting

Cells were washed with PBS (Gibco) and lysed with RIPA buffer (50 mmol/L Tris pH 7.4, 150 mmol/L NaCl, 1% Triton X‐100, 1% sodium deoxycholate, 0.1% SDS, and 1 mmol/L PMSF). After being quantified by Bradford assay, protein samples were separated using a 10% TGX Stain‐Free™ FastCast™ Acrylamide Kit and blotted to Immobilon® NC membranes. The membranes were blocked (5% BSA in TBS‐T) and incubated with primary and secondary antibodies (suggested concentrations in 5% skimmed milk) in sequence. Photographs were taken for chemiluminescence using a ChemiDoc MP Imaging System (Bio‐Rad).

### Colony formation assays

Cells were seeded into six-well plates (0.5–1.0 × 10^4^ cells per well) and cultured in the presence of the indicated intervention. For each cell line, cells cultured under different conditions were fixed with 4% paraformaldehyde (in PBS) at the same time. Afterward, cells were stained with 0.1% crystal violet (in water). A ChemiDoc MP Imaging System (Bio‐Rad) was used for photographing.

### CCK-8 cell viability assay

Cells were cultured and seeded into 96-well plates at a density of 500–1000 cells per well (24 h later, drugs were added at the indicated concentrations). Ten microliters of CCK-8 solution (Yeasen Biotech) were added to each well (100 μL), and then the cells were incubated for 1–4 h at 37 °C. Cell viability was assessed by measuring the fluorescence emission at 450 nm.

### FxCycle ^TM^ propidium iodide (PI)/RNase staining

PCa cells were harvested and fixed with 70% ethanol for 8–10 h at 4 °C. Then, the cells were washed with PBS, and all fixative was removed. Each prepared flow cytometry sample contained at least 0.5 × 10^6^ cells in suspension. Then, 400 μL FxCycle™ PI/RNase Staining Solution (0.5 mL; lot#: 2165130, Invitrogen) was added to each flow cytometry sample and mixed well, and the sample was incubated for 15–30 minutes at room temperature. Cell cycle arrest in the form of a PI signal was detected via NovoCyte Flow Cytometer Systems (ACEA Biosciences).

### Annexin V (AV)-FITC apoptosis detection assay

For the detection of cell apoptosis, cells were harvested by trypsin without ethylenediaminetetraacetic acid (EDTA) and washed with PBS. Then, 380 μL of 1 × binding buffer was added to each sample containing at least 0.5 × 10^6^ cells. Then, AV-FITC apoptosis detection assay reagent (lot#: 185174000, Invitrogen; 10 μL of FITC annexin V and 5 μL of the PI working solution) was added to each sample for 15–30 min at room temperature. Apoptotic cells were analyzed based on AV and PI signals via NovoCyte Flow Cytometer Systems (ACEA Biosciences).

### EU staining

Cells were treated with DMSO or THZ531 for 6 h and cultured in RPMI-1640 medium containing 500 μM EU for 2 h at 37 °C before fixation. EU was detected with a Cell-Light EU Apollo 488 instrument (Cat# C10316–3; lot# S1106, Ribo Biotech) according to the manufacturer’s protocol.

### Sphere-formation assay

Cells were seeded into six-well nonadherent plates (1000 cells per well) and cultured in the indicated dose of THZ531 with DMEM/F-12 (Gibco) containing 50 mg/mL insulin (Sigma), 2.5 mg/mL hydrocortisone (Topscience), 2% B27 (Invitrogen), 0.5 mg/mL epidermal growth factor (EGF), and 0.1 mg/mL basic fibroblast growth factor (bFGF) (2.0 mL per well) at 37 °C. Cultures were added 300 μL (containing equal-concentration of THZ531) every 3 days. The number and sizes of oncospheres were measured at 7th day.

### RNA-Sequencing (RNA-Seq) assay

LNCaP cells (1.0 × 10^6^) were cultured with regular RPMI-1640 medium containing 10% FBS. After being treated with DMSO, 0.2, 0.5, and 1.0 µM THZ531 for 6 h, the cells were trypsinized and collected by centrifugation at 1000 × *g* for 1.5 min at 4 °C. Cells were then washed once with cold 1× PBS and centrifuged at 1000 × *g* for 1.5 min at 4 °C. After discarding the supernatant, total RNA was extracted from LNCaP cells using Hipure RNA Mini Kit (Magen). RNA samples were sequenced using the standard Illumina protocol to create raw sequence files (.fastq files) at LC Sciences. Significant hits were selected based on the following cutoffs: 1 for the log2 fold change and 0.05 for the permutation *p* value. The GO, KEGG, and GSEA analysis were evaluated by bioinformaticists at LC Sciences.

### Cleavage under targets and tagmentation (CUT&Tag) assay

The CUT&Tag assay was performed using the NovoNGS® CUT&Tag 2.0 High-Sensitivity Kit (NovoProtein, N259-YH01). 5.0 × 10^5^ LNCaP cells were washed twice with 1.5 mL of wash buffer and then mixed with activated concanavalin A beads. After successive incubations with the primary antibody (H3K27ac, 4 °C, 16 h) and secondary antibody (RT, 1 h), the cells were washed and incubated with pAG-Tn5 for 1 h. Then, MgCl_2_ was added to activate tagmentation for 1 h. The tagmentation reaction was stopped, and the chromatin complex was digested with a solution containing 10 µL of 0.5 M EDTA, 3 µL of 10% SDS and 2.5 µL of 20 mg/mL Proteinase K at 55 °C for 2 h. The transposed DNA fragments were purified using a Qiagen MinElute PCR Purification Kit and amplified using NEBNext Ultra II Q5 Master Mix (New England Biolabs, M0544L). The libraries were sequenced by Novogene using the Illumina NovaSeq 6000 platform.

The trimmed sequencing reads (trim_galore) were aligned to the reference human genome (GRCh37/hg19) using HISAT2 with X, no-spliced-alignment, and no-temp-splicesite parameters. PCR duplicates were removed using picard MarkDuplicates (doi:10.1101/gr.107524.110). Peaks were called using MACS2 and annotated using HOMER. A differential expression analysis was performed using the DiffBind package (v3.0.11), and a heatmap was generated using deeptools (doi:10.1093/nar/gkw257). A motif enrichment analysis and GREAT analysis were performed using HOMER and GREAT, respectively.

### RNA isolation and RT-qPCR

Cells were harvested and extracted for RNA using Hipure RNA Mini Kit (Magen) according to the manufacturer’s instructions. cDNA templates were synthesized using a Reverse Transcription Kit (with dsDNase) (Cat# BL699A, Biosharp). RT-qPCR assays were performed using a Bio-Rad CFX96 thermocycler (Applied Bio-Rad CFX Maestro). The relative mRNA levels of the indicated genes were normalized to the level of GAPDH mRNA. The primer sequences for assays using ChamQ^TM^ SYBR qPCR master mix (Vazyme Biotech) are listed in Supplementary Table 1.

### Statistical analysis

GraphPad Prism 8.0 was used for statistical calculations. Statistical significance was evaluated using two-sided unpaired t-tests. In the figures with bar graphs, the values are presented as the means ± SDs. *p* value ≤ 0.05 were considered statistically significant: *** indicates *p* ≤ 0.001, ** indicates *p* ≤ 0.01, and * indicates *p* ≤ 0.05.

## Results

### CRISPR/Cas9 screening identifies CDK12 as a conservative kinase target of PCa

**Fig. 1:**
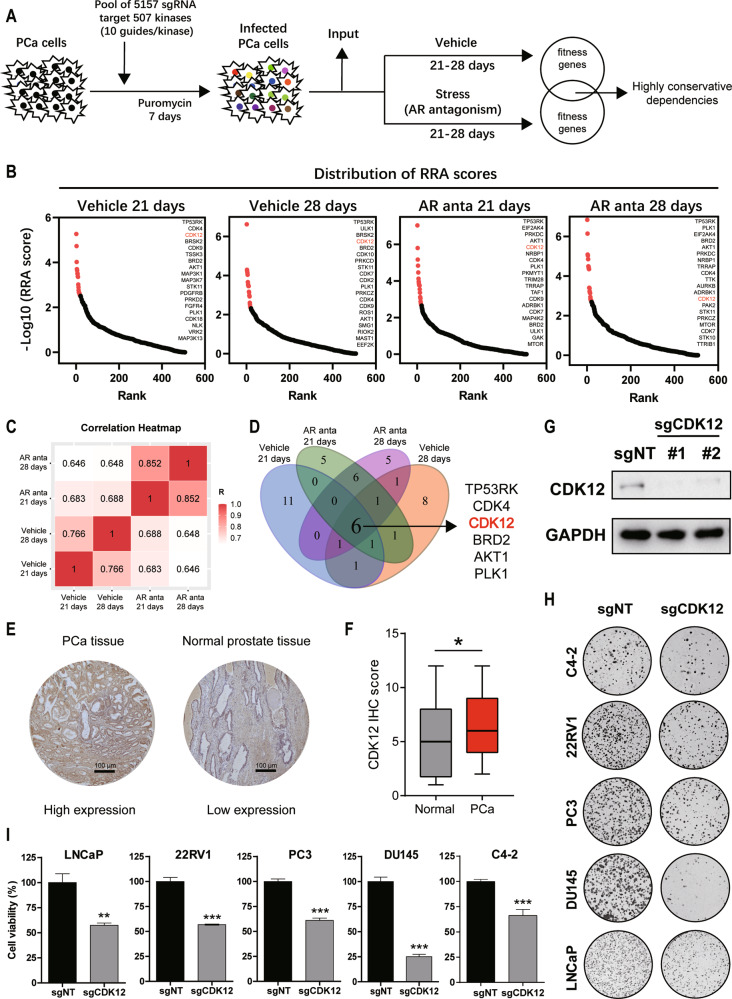
CRISPR/Cas9 screening to identify genes critically required for PCa. **A** Schematic illustration of CRISPR/Cas9 screening to identify conserved kinases in C4–2 cells cultured with normal or 10–25 μM enzalutamide. **B** RRA scores for the kinome. The top 20 candidate genes are marked in red. **C** Heatmap showing the correlation of the kinome among the normal and AR antagonism groups on the 21st and 28th days. **D** Venn diagram showing the overlap of the top 20 candidate genes from each group. **E** Typical images of immunostaining of the CDK12 protein in PCa and normal prostate tissues. The scale bars represent 100 µm. **F** IHC score of the CDK12 protein levels in PCa and normal prostate tissues. **G** Level of CDK12 knockout in PCa cells, as measured by western blotting. **H** PCa cell proliferation assessed by the colony formation assay. **I** PCa cell viability assessed by the CCK-8 cell viability assay.

To identify the highly conservative dependencies of CRPC as the most promising target, we performed a CRISPR screen targeting 507 kinases to detect genes critically required for PCa cells under normal conditions or under enzalutamide-treated culture conditions (Fig. 1A). The top 20 candidate genes in each group were commonly enriched in cell cycle, transcription, and DDR regulation (Fig. 1B). The correlation heatmap presented better similarity between the 21st and 28th days of the same intervention groups, suggesting a reasonable screening result (Fig. 1C). We further overlapped the top 20 candidate genes from each group and identified six kinases that were depleted in both normal- and enzalutamide-cultured PCa cells (Fig. 1D). Among them, CDK4, BRD2, AKT1, and PLK1 have been validated as critical kinases of PCa.

We focused on CDK12, a key regulator of transcription elongation and DDR, in follow-up experiments. To exclude the screening bias caused by a single cell line, we also validated the dependencies of LNCaP, 22RV1, and DU145 cells on CDK12 from the Project Score database (Fig. S1A). To further investigate the function of CDK12, we identified significantly higher CDK12 mRNA levels in PCa than in normal prostate tissues from the PCa database of the Tomlins cohort (Fig. S1B). The Cancer Genome Atlas (TCGA) data showed that PCa patients with lower CDK12 mRNA levels experienced slightly longer DFS (Fig. S1C). Next, we analyzed CDK12 protein levels using a TMA containing 150 PCa specimens by immunohistochemical analysis and found that CDK12 protein was expressed at significantly higher levels in PCa tissues (Fig. 1E, F), indicating the feasibility of CDK12 as a target for PCa therapy.

To further validate our findings, we infected a panel of PCa cells with CDK12 sgRNAs. Genetic depletion of CDK12 (Fig. 1G) inhibited the proliferation and viability of both hormone-sensitive prostate cancer (HSPC) and CRPC cell lines in clone formation (Fig. 1H) and CCK-8 cell viability assays (Fig. 1I), respectively. Together, our data indicate that CDK12 is conservatively required for PCa cells.

### CDK12 inhibition shows powerful antineoplastic properties against PCa cells

**Fig. 2:**
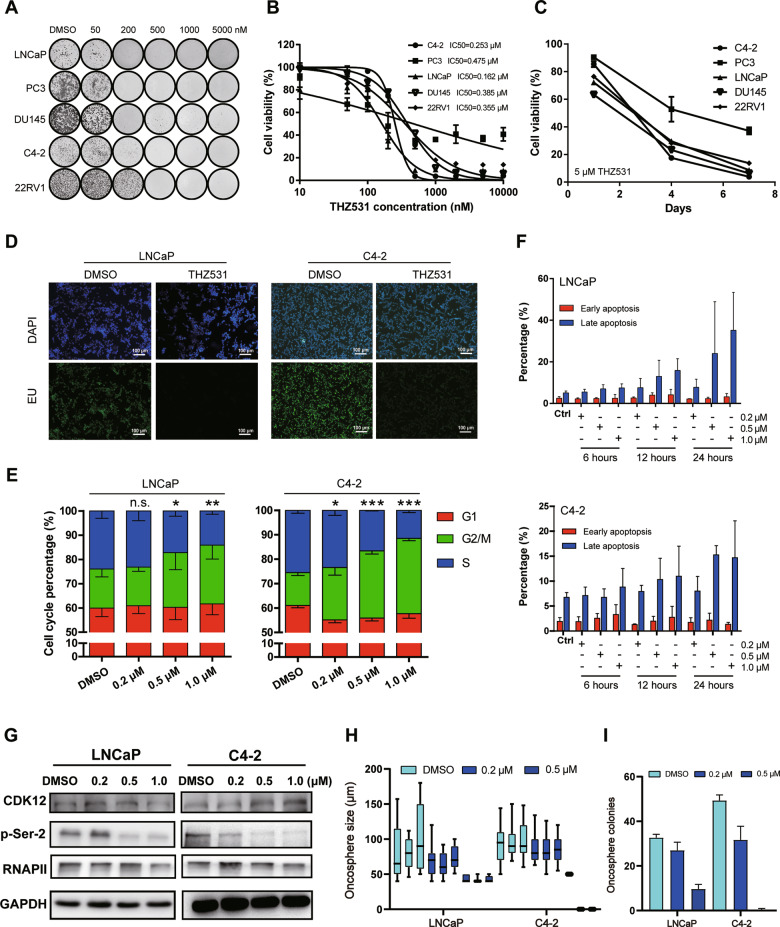
CDK12 inhibition induces apoptosis in PCa cells. **A** PCa cells were seeded and treated with THZ531 in colony formation assays. **B**, **C** CCK-8 cell viability assay of PCa cells treated with the indicated time and concentrations of THZ531. **D** EU incorporation assay of LNCaP and C4–2 cells showing the difference in newly transcribed RNA after treatment with 1.0 µM THZ531 for 6 h. The scale bars represent 100 µm. **E** FACS analysis of the LNCaP and C4–2 cell cycle after THZ531 treatment for 12 h. **F** FACS analysis of LNCaP and C4–2 cells stained with AV and PI after THZ531 treatment for the indicated times. **G** Western blotting showing the level of RNAPII CTD S2 phosphorylation in LNCaP and C4–2 cells treated with THZ531. **H** The numbers of oncosphere colony in LNCaP and C4–2 cells treated with DMSO or 0.2, 0.5 µM THZ531 for 7 days. **I** The sizes of oncospheres observed for LNCaP and C4–2 cells treated with DMSO or 0.2, 0.5 µM THZ531 for 7 days.

We further treated the panel of PCa cells with THZ531 (a covalent inhibitor of CDK12) [22]. Colony formation assays demonstrated that THZ531 substantially inhibited cell proliferation (Fig. 2A). CCK-8 assays revealed a concentration- and time-dependent decrease in PCa cells viability upon THZ531 treatment (Fig. 2B, C). To intuitively investigate the effect of CDK12 inhibition on transcription, we treated LNCaP and C4–2 cells with THZ531 and observed a conspicuous reduction in the amount of newly transcribed RNA using fluorescently labeled EU incorporation assays (Fig. 2D). FACS cell cycle assays with escalating doses of THZ531 displayed an increasing number of cells in the sub-G2/M phase (Fig. 2E). We subsequently used flow cytometry to assess AV and PI staining in apoptotic cells and observed dose- and time-dependent enhancement in PI signals of positively stained cells (Fig. 2F), indicating that CDK12 inhibition induces apoptosis of PCa cells. As previously reported [22], a dose-dependent decrease in the phosphorylation level of S2 of the RNAPII CTD was present in THZ531-treated PCa cells (Fig. 2G). In addition, the sphere-formation assays demonstrated the lower sphere-formation efficiency on the THZ531-treated LNCaP and C4–2 cells, including a gradual decrease in the expansion sizes (Fig. 2H, S1D) and the total number (Fig. 2I, S1D) of primary spheres formed over time for a given number of total tumor cells, suggesting the impact of THZ531 on the sphere-forming ability of PCa cells.

### CDK12-ISTs contain prostate lineage-specific genes and contribute to the survival processes of PCa cells

**Fig. 3:**
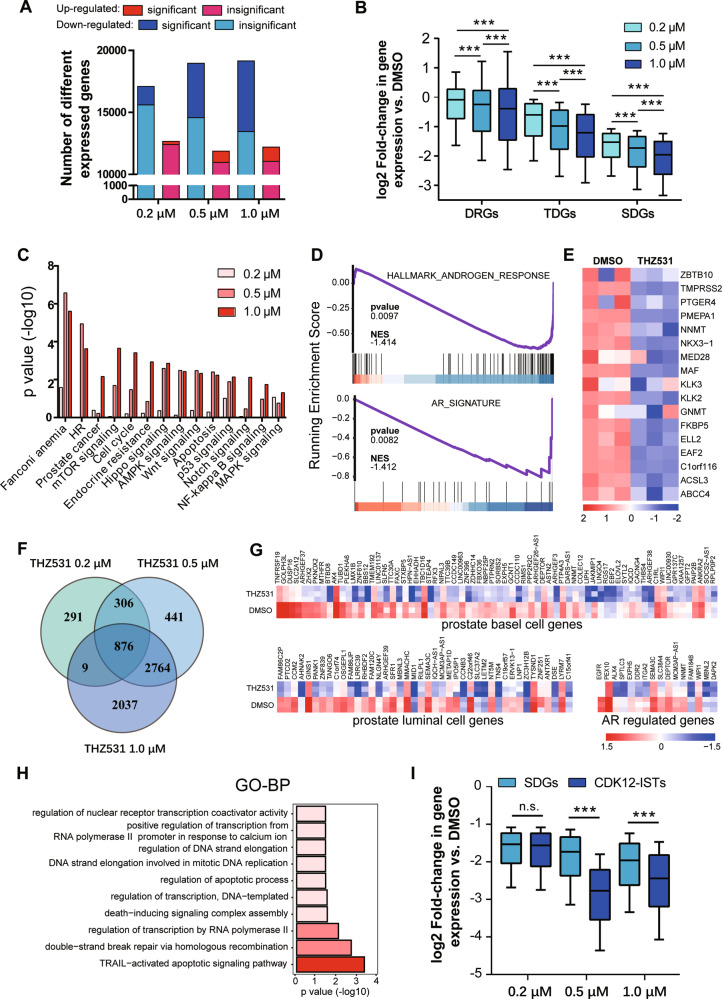
CDK12-ISTs contribute to the survival processes of PCa cells. **A** The numbers of differentially expressed genes (DEGs) induced upon either DMSO or THZ531 treatment in LNCaP cells at 6 h. **B** Log2 fold changes in DEGs, total downregulated genes (TDGs) and significantly downregulated genes (SDGs) expression induced by THZ531 in LNCaP cells for 6 h. **C** KEGG analysis of significant DEGs in LNCaP cells treated with THZ531 for 6 h. **D** GSEA revealed that gene sets of androgen response and AR signature were significantly downregulated in THZ531-treated LNCaP cells. **E** Heatmap showing the downregulation of AR score genes in THZ531-treated LNCaP cells. **F** Venn diagram showing the SDGs among different doses of THZ531 as CDK12-ISTs. **G** Heatmap showing the downregulation of prostate basal/luminal cell genes and AR-regulated genes related to CDK12-ISTs. **H** Enriched GO functional categories of CDK12-ISTs. **I** Log2 fold changes in the mRNA abundance of CDK12-ISTs and SDGs.

To examine the effect of CDK12 inhibition on the transcriptome, we performed RNA-Seq on THZ531-sensitive LNCaP cells. Samples were treated with DMSO or THZ531 at 0.2/0.5/1.0 µM for 6 h, excluding the impact of cell cycle arrest (Fig. S2A). THZ531 resulted in a global- and concentration-dependent reduction in steady-state mRNA (Fig. 3A, B). Notably, the transcriptome alteration mediated by CDK12 inhibition was inconsistent with that of the CDK12 loss mutation [19] (Fig. S2B, C), which reveals underlying mechanism differences between them.

In addition to the expected enrichment of the DDR pathway (Fig. S2D), CDK12 inhibition also suppressed key signaling associated with the progression and drug resistance of PCa, such as the Wnt [23], Hippo [24] and, Notch pathways [25] (Fig. 3C). The gene sets HALLMARK_ANDROGEN_RESPONSE and AR_SIGNATURE [26] were significantly de-enriched upon THZ531 treatment (Fig. 3D). Downregulation was also found in AR score genes [27] (Fig. 3E). We validated the significantly lower mRNA levels of canonical AR targets in THZ531-treated LNCaP cells (Fig. S2E). These findings may partially explain the dependence of PCa cells on CDK12 under AR antagonism.

Prior studies have proven that inhibiting transcription preferentially inactivates lineage-specific or oncogenic genes related to tumor survival [28–32]. Based on the essential transcription function of CDK12, we hypothesized that transcripts sensitive to CDK12 inhibition may also include lineage-specific and oncogenic transcripts and, rather than being random, contribute biological survival relevance to PCa. Then, we identified a distinct class of 867 transcripts that were significantly downregulated in a dose-independent fashion (Fig. 3F) and termed this group CDK12 inhibition-sensitive transcripts (CDK12-ISTs). As expected, CDK12-ISTs contained a number of prostate lineage-specific genes, including basal/luminal [33] and AR-regulated genes (Fig. 3G), suggesting tissue specificity of CDK12-ISTs. More importantly, the GO analysis revealed the participation of CDK12-ISTs in basic survival processes, such as transcription, DDR, and apoptosis regulation, highlighting the critical dependences of PCa on CDK12 (Fig. 3H). In addition, CDK12-ISTs were actually more sensitive to THZ531 treatment than other affected transcripts (Fig. 3I), further supporting our hypothesis.

### Integrating SE landscapes with CDK12-ISTs indicates potential lineage-specific oncogenes of PCa cells

**Fig. 4:**
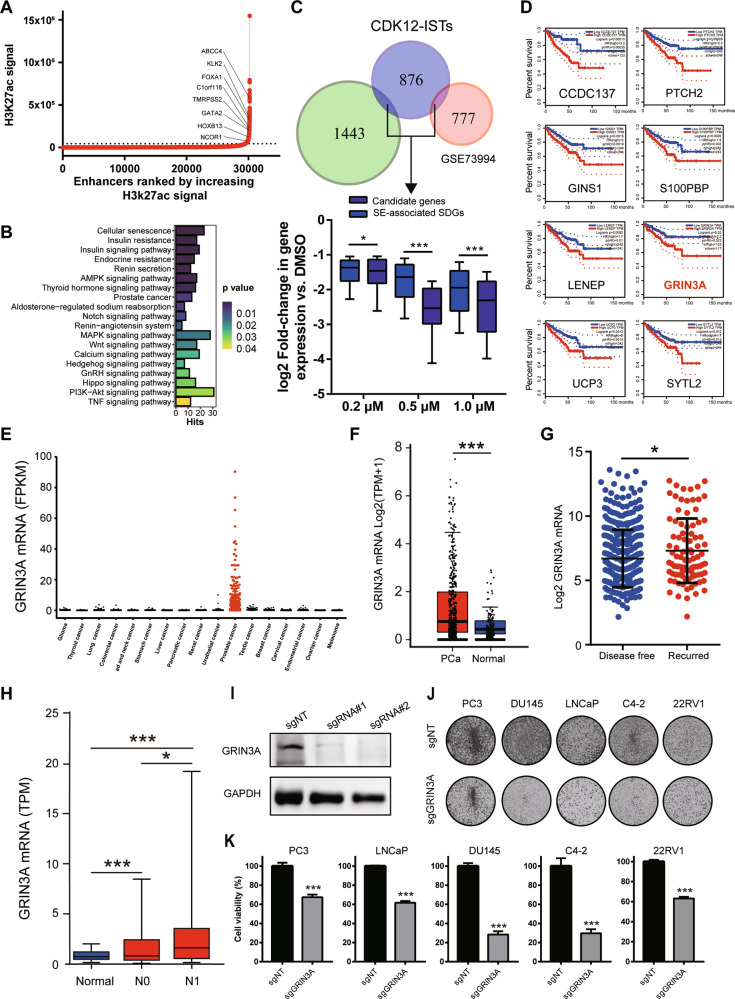
SE-driven CDK12-IST enumerates oncogenes. **A** Hockey stick plots showing input-normalized, rank-ordered H3K27ac signals and SE-associated genes. **B** KEGG analysis of SE-associated genes. **C** Venn diagram showing the candidate genes between CDK12-ISTs and SE-associated genes (top). Log2 fold changes of SE-driven CDK12-ISTs compared to significantly downregulated SE-associated genes (bottom). **D** Kaplan–Meier curves indicating the associations between the mRNA levels of eight candidate genes and DFS in patients with PCa in TCGA database. **E** GRIN3A mRNA levels in various types of human cancer. **F** GRIN3A mRNA levels in PCa tissues and normal prostate tissues. **G** GRIN3A mRNA levels in remission and recurrent PCa. **H** GRIN3A mRNA expression in primary PCa and PCa with regional lymph node metastasis. N0: no regional lymph node metastasis; N1: metastases in 1 to 3 axillary lymph nodes. **I** The effectiveness of GRIN3A knockout in PCa cells was measured by western blotting. **J**, **K** PCa cell proliferation and viability were assessed by a colony formation assay and CCK-8 cell viability assay, respectively.

SEs regulate specific gene expression programs to sustain fundamental cell biology, including key lineage-specific oncogenes that control the cancer cell state [34, 35], which is highly relevant to the characterization of CDK12-ISTs. To characterize SE-associated transcripts in PCa, we performed CUT&Tag assays in LNCaP cells using an antibody recognizing H3K27ac modification (Fig. 4A) and obtained 1443 SE-associated genes involved in the progression and drug resistance of PCa (Fig. 4B, C), including essentially lineage-specific transcription factors, such as FOXA1, GATA2, HOXB13 [36], and NCOR1 [37] (Fig. 4A).

A few SE-driven genes are especially vulnerable to transcriptional defects and thereby serve as (i) the reason conferring the sensitivity of cancer to transcription inhibition and (ii) cancer-type-specific targets [28–32]. Hence, we hypothesized that SE-driven CDK12-ISTs may be enumerated as candidate oncogenes and further contribute to the essential dependence of PCa cells. Therefore, we integrated SE-associated genes (including the cohort stimulated with R1881; GSE73994) with CDK12-ISTs, resulting in 53 overlapping genes (Fig. 4C and S2F), which were expectedly more intensely repressed by THZ531 (Fig. 4C), representing a set of SE-driven genes addicted to transcription mediated by CDK12. Among them, numerous genes have been considered potential oncogenes in PCa (EGFR, DDR2, and RLN2) [38–40]. While we identified eight more genes with low mRNA levels that were associated with superior DFS in patients with PCa (Fig. 4D). RT-qPCR validated the reduction in their mRNA levels after THZ531 treatment (Fig. S2G).

To further validate the oncogenic function of SE-driven CDK12-ISTs, we focused on GRIN3A, which encodes a subunit of N-methyl-D-aspartate receptors (NMDARs) [41, 42] and shows a lineage-specific expression pattern in PCa (Fig. 4E). GRIN3A was significantly upregulated in PCa (Fig. 4F) compared to normal prostate tissues, then patients with high expression of GRIN3A exhibited a higher recurrence risk (Fig. 4G) and worse lymphatic metastasis (Fig. 4H). Knockout of GRIN3A (Fig. 4I) significantly inhibited the proliferation and viability of PCa cells (Fig. 4J, K). Thus, SE-driven CDK12-ISTs may function as lineage-specific oncogene and contribute to the vulnerability mediated by CDK12 inhibition.

### CDK12 inhibition strikingly synergizes with AR antagonism via epigenetic alteration of AR signaling

**Fig. 5:**
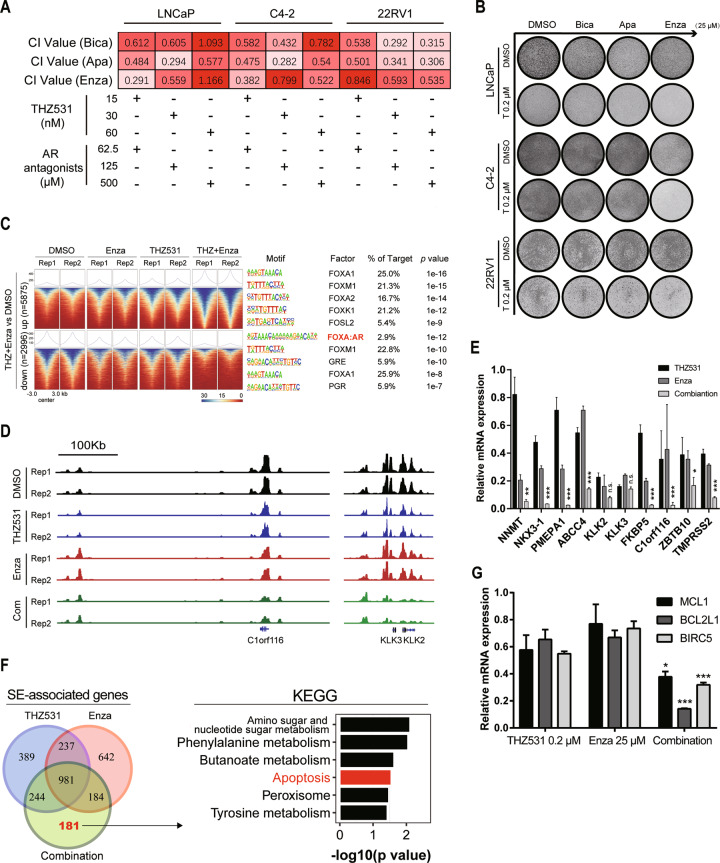
THZ531 synergizes with AR antagonists in PCa cells. **A** Synergistic relationship between different doses of THZ531 and AR antagonists in the CCK-8 assay. CI values < 1 represent synergy. **B** PCa cells treated with 0.2 µM THZ531, 25 µM AR antagonists or combination in the colony formation assay. **C** Heatmap showing the signal intensity of H3K27ac and motif enrichment in treated LNCaP cells. **D** IGV views of sequencing data at AR target loci under different conditions. **E** Relative mRNA expression of canonical AR target genes in LNCaP cells treated for 24 h. **F** Venn diagram with the number of SE-associated genes among treated LNCaP cells and KEGG analysis of SE-associated genes exclusive to the combination group. **G** Relative mRNA expression of MCL1, BIRC5, and BCL2L1 in LNCaP cells treated for 24 h.

Based on the CRISPR screen result under AR antagonism and the impact of THZ531 on AR signaling, we hypothesized that CDK12 inhibition may synergize with AR antagonism. We treated PCa cells with THZ531 and first- (bicalutamide) or next-generation AR antagonists (apalutamide and enzalutamide), and found synergy with no limitation to a single antagonist or cell line (Fig. 5A). To better imitate the real dose of AR antagonists administered to patients [43, 44], we treated PCa cells with 25 µM AR antagonists, and found that a low dose of 0.2 µM THZ531 was enough to induce striking synergy in colony formation assays (Fig. 5B), supporting the essential requirement of CDK12 for PCa cells under AR antagonism.

We next performed CUT&Tag assays of H3K27ac modification in treated LNCaP cells (0.2 µM THZ531, 25 µM enzalutamide and their combination for 24 h) compared to the control group. We observed remarkable differences in H3K27ac signals among different treatments (Fig. 5C and S3A). The FOXA:AR motif was significantly enriched in decreasing H3K27ac signal regions in the combination group (Fig. 5C), explaining the suppressive AR score genes in the transcriptome. Genes with significantly declining H3K27ac signals were enriched in the PCa pathway in the combination group, suggesting attenuated PCa activity (Fig. S3B). A similar H3K27ac signal drop-off in the combination group was also found in AR targets (Fig. 5D and S3C), and mRNA levels presented a dramatic decline in the combination group (Fig. 5E). These results reveal that combination treatment represses AR signaling by inducing attenuated H3K27ac signaling.

According to the analysis of H3K27ac modification, SE-associated genes were more susceptible to treatments than typical enhancers (TEs) (Fig. S3D), implying an important role of SE-associated genes in synergy. We next identified that 181 SE-associated genes specific to the combination group were enriched in the apoptosis pathway (Fig. 5F). The KEGG analysis of the SE-associated genes also demonstrated the most significant enrichment of the apoptosis pathway in the combination treatment (Fig. S3E). Flow cytometry also detected more apoptotic cells after combination treatment (Fig. S3F). RT-qPCR validated prominent decreases in MCL1, BIRC5, and BCL2L1 (anti-apoptosis genes) mRNA levels in the combination group (Fig. 5G). Notably, BIRC5 and BCL2L1 are two of three key hub target genes that contribute to PCa carcinogenesis and progression [45].

## Discussion

Somatic loss-of-function CDK12 mutations generate an aggressive subtype of CRPC with poor outcomes [19–21]. While we identified CDK12 as critically required for PCa cell survival via a CRISPR screen. Both genetic deletion and functional inhibition suggested that CDK12 accelerates PCa progression to a certain extent, similar to that in other malignancies [16–18]. The inconsistent transcriptome alteration further revealed discrepant mechanisms between CDK12 inhibition and somatic loss-of-function CDK12 mutations in clinical patients. Our experiments demonstrated that HSPC and CRPC cells both respond dramatically to THZ531, highlighting the reliability of CDK12 as a PCa druggable target.

Cancer cells require highly active transcription to maintain their essential and oncogenic biological functions, including rapid proliferation and aggressive invasion [46]. Therefore, it is reasonable that CDK12-IST-associated survival pathways may primarily contribute to the vulnerability of PCa cells to CDK12. To our surprise, CDK12-ISTs consisted of genes related to prostatic identity and AR-regulated genes, implying the cancer-type-specific feature of CDK12-ISTs.

SEs control specific gene expression programs [34, 35], including the expression of a few critical oncogenes, which are particularly vulnerable to transcription defects [28–32]. By virtue of this theory, studies [28–32] have successfully identified oncogenes by inducing transcription initiation defects. Therefore, we hypothesized that SE-driven CDK12-ISTs may enumerate oncogenes that contribute to the dependencies of PCa cells. Subsequent integrative results of the SE landscape and CDK12-ISTs validated our hypothesis. It is worth noting that many AR-regulated genes can be found in SE-associated genes without androgen. Therefore, we believe that oncogenes under androgen stimulation (similar to the early stage of PCa) may also be suppressed by CDK12 inhibition and thus impact PCa cell survival. For this reason, we integrated CDK12-ISTs and SE-associated genes, which included a cohort stimulated by R1881.

Based on our CRISPR results and the impact of THZ531 on AR signaling, we hypothesized and validated a synergistic effect between THZ531 and AR antagonists. To our excitement, the synergetic outcome was not restricted to HSPC or CRPC cells or to first- or next-generation AR antagonists. Mechanistically, the FOXA:AR motif was significantly enriched with a decreasing H3K27ac signal after combination treatment, strongly supporting that combination treatment results in hypoactive AR transcription activity, which is consistent with transcriptome alteration.

The KEGG analysis of SE-associated genes in combination treatment demonstrated the most significant enrichment of the apoptosis pathway, which contains important anti-apoptosis genes BIRC5 and BCL2L1. AR binds to agonist-liganded ARBEs to upregulate the hub target genes BIRC5 and BCL2L1, contributing to PCa carcinogenesis and progression [45]. In addition, the downregulation of BIRC5 is considered to be one of the essential mechanisms of enzalutamide-induced inhibition of PCa cells [45, 47]. Thus, significant downregulation at the BIRC5 and BCL2L1 mRNA levels may be partly responsible for the synergistic effect.

In summary, we used a CRISPR screen and identified CDK12 as a conservative vulnerability of PCa cells. The CDK12 inhibitor THZ531 presented an obvious anti-PCa effect. CDK12-ISTs contribute to survival processes, and the preferential downregulation of SE oncogenes may explain the anti-PCa properties of CDK12 inhibition. The synergy between THZ531 and AR antagonists may be driven by attenuated H3K27ac signaling on AR targets and intensive SE-associated apoptosis pathways, further suggesting the druggable value of CDK12 for PCa therapy.

## Supplementary information

Figure 1S

Figure 2S

Figure 3S

Supplementary Table 1

## Data Availability

The raw data used for the CRISPR screen, RNA-Seq and CUT&Tag samples are available in the Genome Sequence Archive (Genomics, Proteomics & Bioinformatics 2017) database under accession number HRA000724.
