# Supplementary figures and images for: CRISPR screening identifies CDK12 as a conservative vulnerability of prostate cancer
Source: Cell Death Dis. 2021 Jul 27;12(8):740. doi: 10.1038/s41419-021-04027-6 (PMC8316367; doi:10.1038/s41419-021-04027-6)

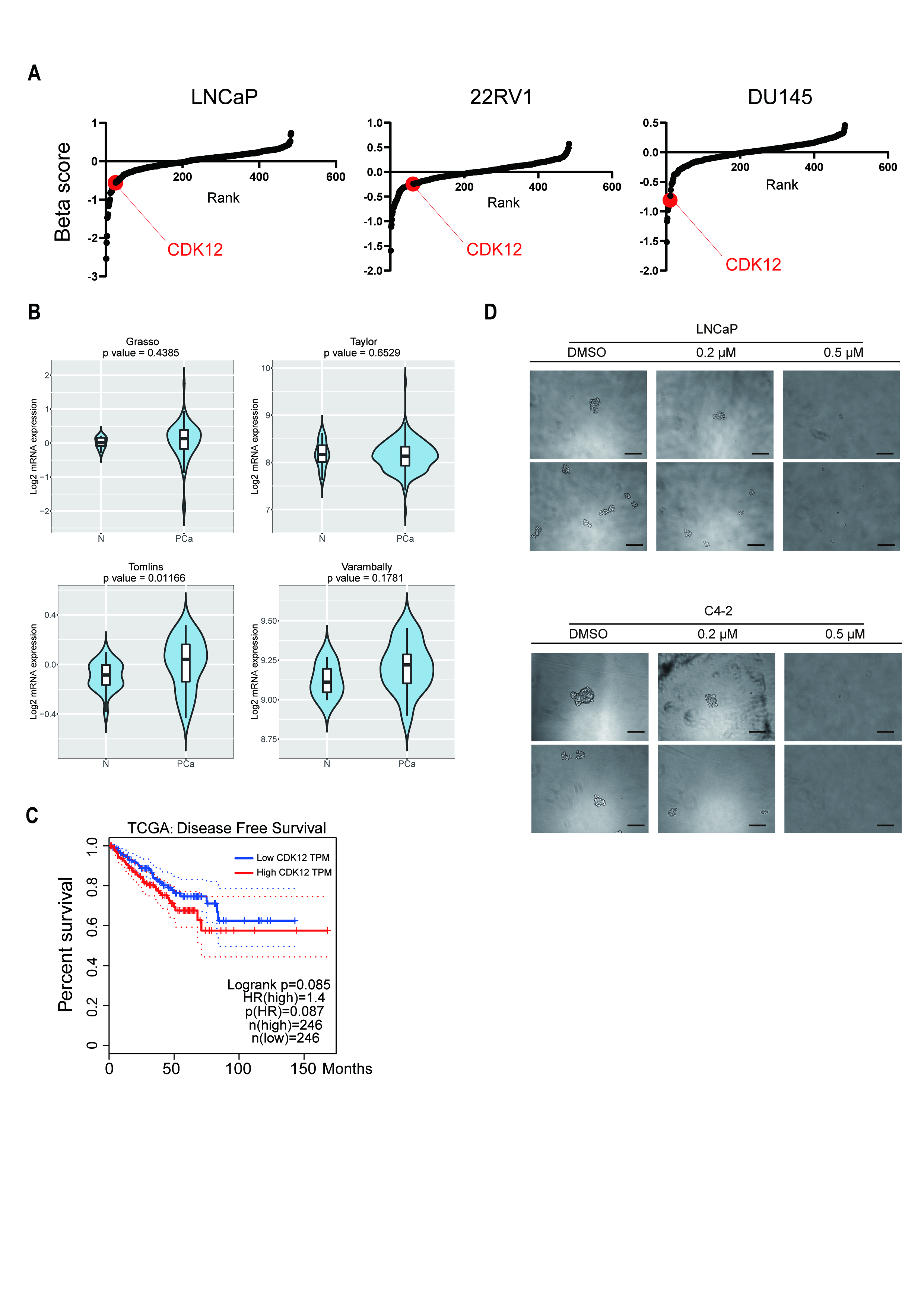

Supplement: Supplementary file 1 — Figure 1S [file 41419_2021_4027_MOESM1_ESM.tif]

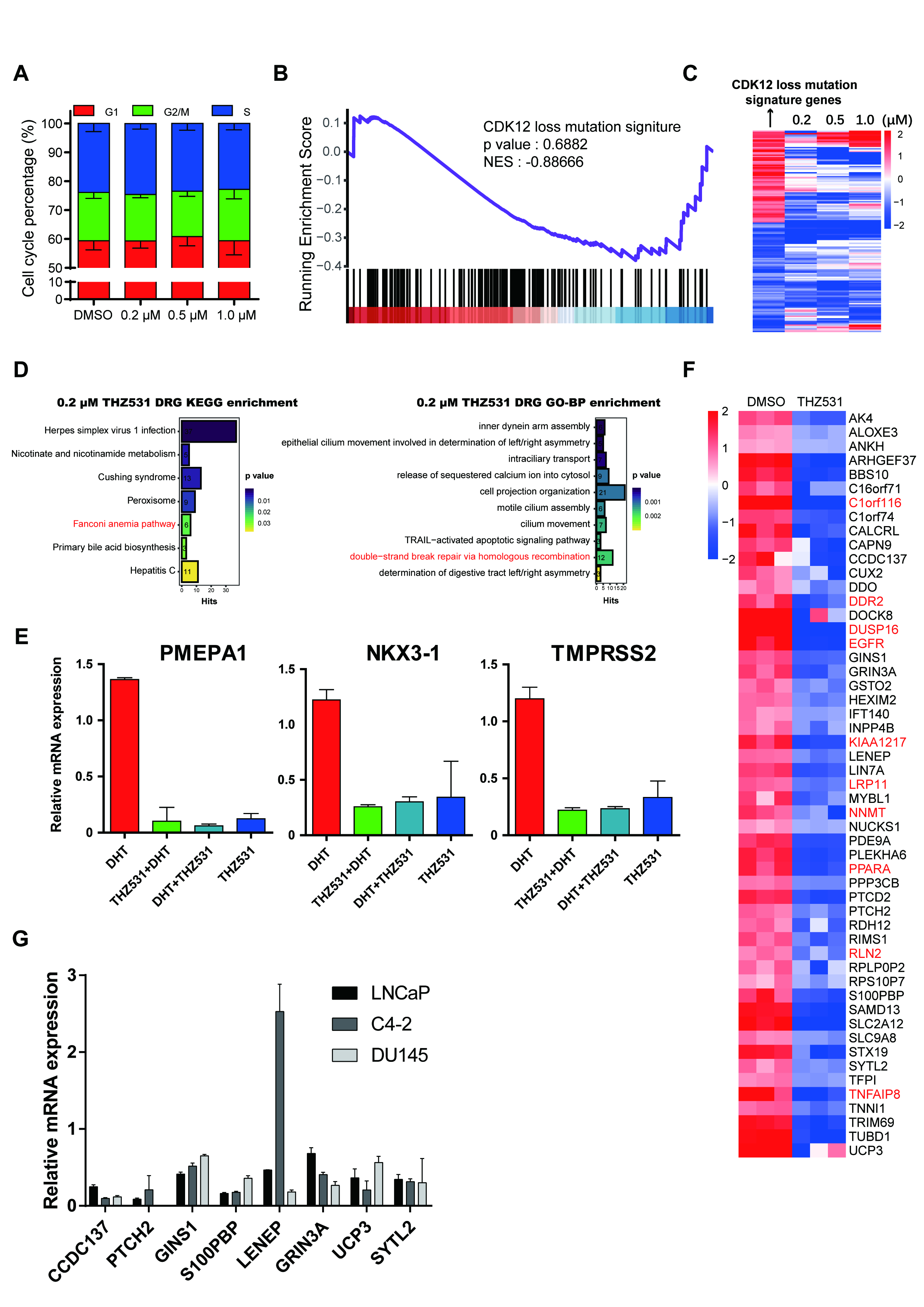

Supplement: Supplementary file 2 — Figure 2S [file 41419_2021_4027_MOESM2_ESM.tif]

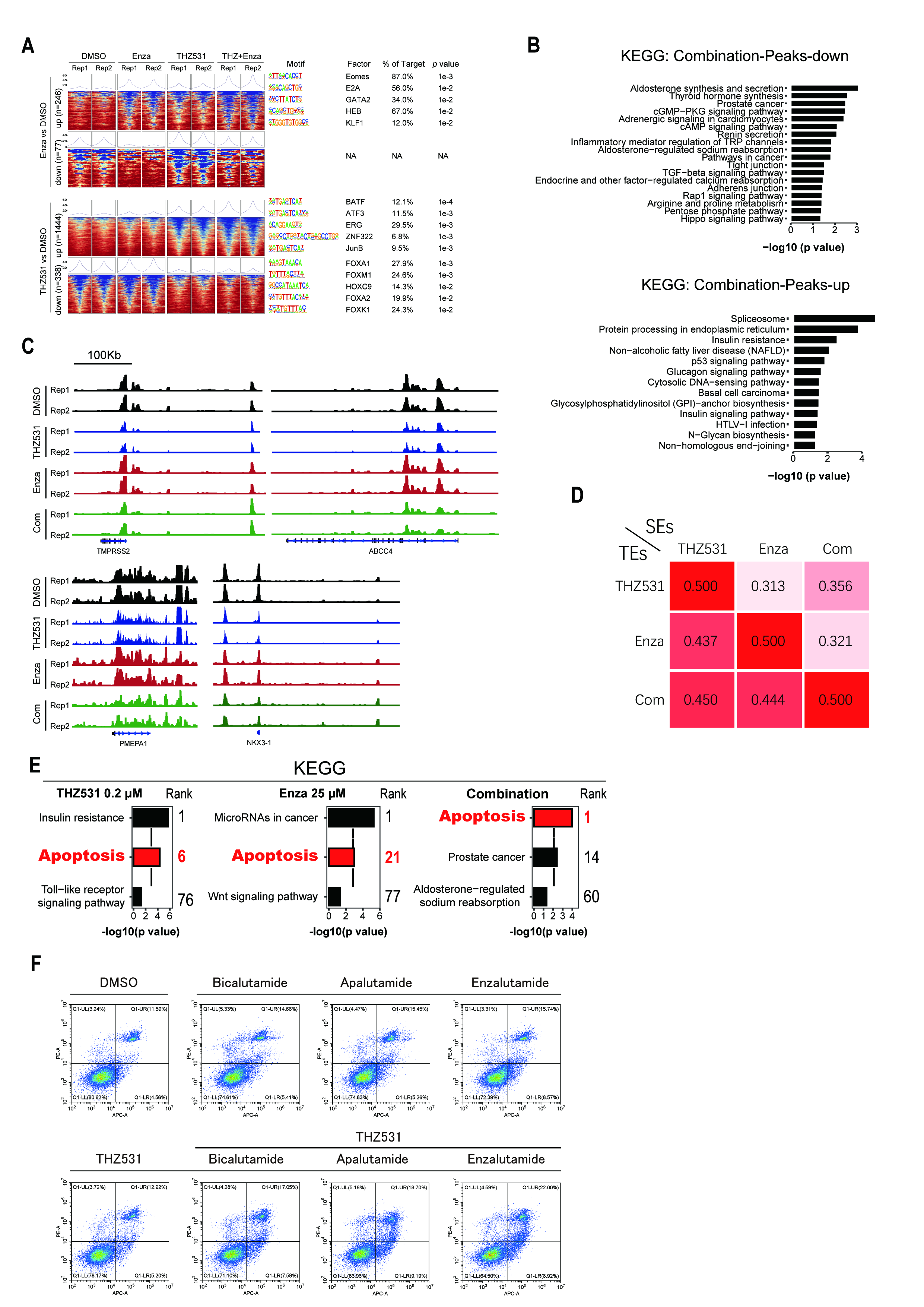

Supplement: Supplementary file 3 — Figure 3S [file 41419_2021_4027_MOESM3_ESM.tif]
